# Early immunotherapy is highly effective in IgG1/IgG4 positive IgLON5 disease

**DOI:** 10.1007/s00415-020-09924-y

**Published:** 2020-05-22

**Authors:** Thomas Grüter, Volker Behrendt, Corinna I. Bien, Ralf Gold, Ilya Ayzenberg

**Affiliations:** 1 Department of Neurology, St. Josef Hospital, Ruhr University Bochum, Gudrunstr. 56, 44791 Bochum, Germany; 2 Laboratory Krone, Siemensstr. 40, 32105 Bad Salzuflen, Germany; 3 Department of Neurology, I.M. Sechenov First Moscow State Medical University, Moscow, Russia

Dear Sirs,

In the pathogenesis of anti-IgLON5 disease, there is evidence for both neurodegenerative and autoimmune mechanisms [1–3]. While strong association to anti-IgLON5-antibodies and distinct HLA haplotypes suggest an active role of inflammatory processes, older age of manifestation, progressive neuronal tauopathy without signs of inflammation, and an association to the *MAPT*-H1 haplotype, are more typical for neurodegenerative diseases [1]. Data on immunotherapy are contradictory and cannot solve this dilemma. Stabilization as well as no therapy effect with lethal outcome have been reported so far [3]. Improvement under immunotherapy is described in a few cases [3, 4].

Here, we present a patient with an acute to subacute bulbar manifestation of anti-IgLON5 disease, mimicking a myasthenic crisis, and a dramatic recovery under immunotherapy started 1 week after disease onset.

An 82-year-old lady suffered for a few days from dysesthesia in her hands and feet, an unsteady gait, and augmented sweating. The patient suffered from a marked fatigue and two until three sleep attacks per day that occurred in passive situations, lasted for 15 until 20 min and did not interfere with her routine activities. Initial clinical examination revealed moderate weakness in cervical flexion and extension (MS 4/5), severe gait ataxia, pronounced dysphagia, dysarthria, hypophonia, and a minimal right-side ptosis, suspicious of myasthenia gravis. Repetitive nerve stimulation as well as brain and spinal MRI were unremarkable. On the second day of admission, she progressed to aphonia, severe dysphagia, and weakness of the neck muscles (MS 3/5). Fiber optic evaluation of swallowing (FEES) revealed a pronounced hypotonic deglutition with penetration and slight aspiration of viscous liquids (Fig. 1a). Under suspicion of a myasthenic crisis, intravenous immunoglobulins (IVIg, 2 g/kg BW) were initiated.

**Fig. 1:**
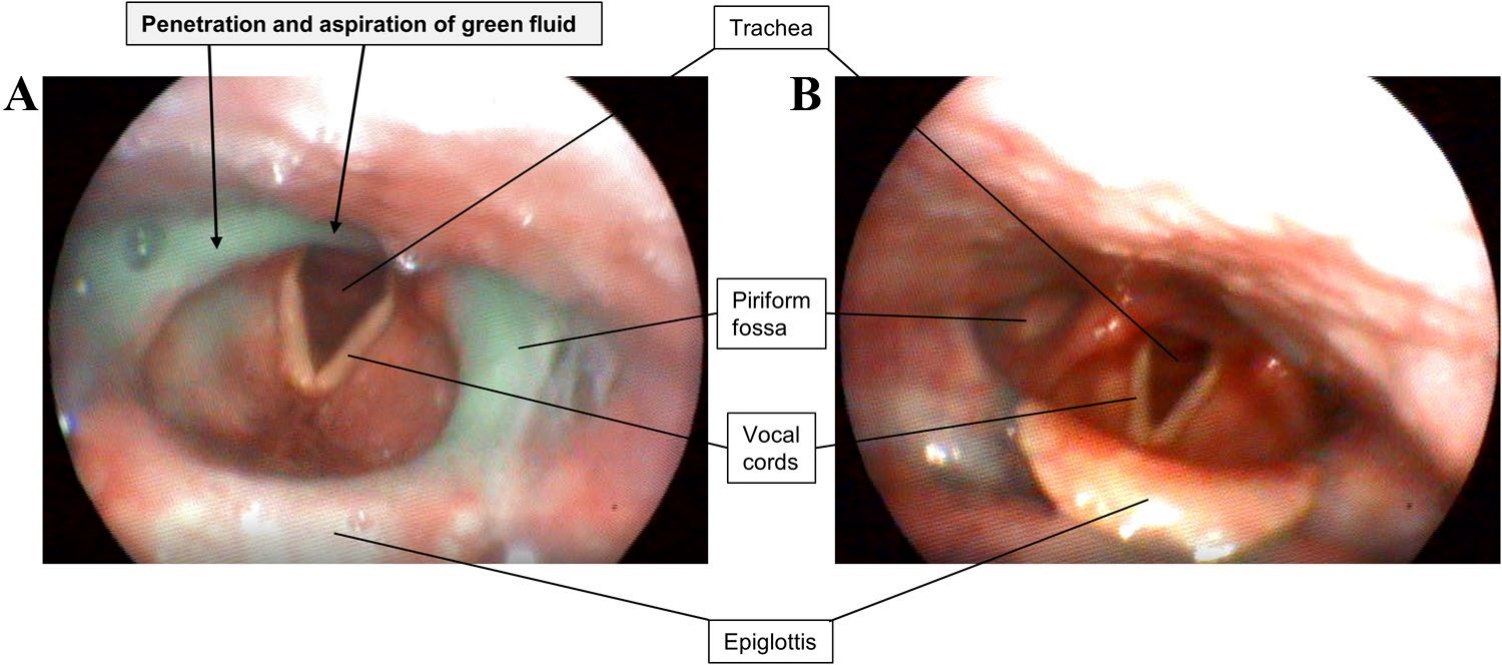
Fiber optic evaluation of swallowing (FEES) before (**a**) and after (**b**) IVIg treatment. Before treatment, green viscous fluid penetrated the laryngeal inlet with slight contact with the vocal folds. After treatment, no penetration or aspiration were evident after swallowing green fluid

During the next 3 days, her condition improved dramatically, she was able to walk without any help and the neck weakness normalized. Furthermore, her voice became clear and repeated FEES showed a striking improvement without further penetration or aspiration (Fig. 1b). A broad serological screening revealed positive anti-IgLON5 IgG (in both tissue based and cell based immunofluorescence, IgG 1:2560, IgG1 1:80, IgG2 1:40, IgG4 1:640, IgG3 negative), whereas antibody testing against acetylcholine-receptor, muscle-specific kinase and titin resulted negative. The patient refused to perform a lumbar puncture. The patient carried the HLA haplotype DQB1*05:01, but not DRB1*10:01. Five weeks later, she was nearly symptom-free and IVIg therapy (1 g/kg every 4 until 6 weeks) was continued.

Today, 1 year after the commencement of the symptoms, the patient is asymptomatic and lives alone without a care service. Despite dramatic clinical recovery of the patient, the serum titer of anti-IgLON5 antibodies did not decline at the last follow-up.

Here, we describe for the first time a patient with anti-IgLON5 disease and a complete recovery under early immunotherapy. In particular, two factors appear to be decisive for the therapy success in this case.

Firstly, IVIg was started within 1 week after disease onset. A slowly progressive disease course and late diagnosis usually result in substantial therapy delay [2, 5]. In line with this, previously reported therapy effects were less obvious and some authors doubted if an immunosuppressive treatment would be effective in this disease [1, 2]. However, subacute disease progression has been reported in a few other cases, probably representing a favorable timepoint for immunotherapy initiation [5–7]. Interestingly, up to 20% of patients have a relative rapid clinical presentation, in less than 4 months.

Secondly, we identified both IgG4 and IgG1 anti-IgLON5 antibodies in our patient. Anti-IgLON5-IgG1 (but not IgG4) cause an irreversible internalization of surface IgLON5 in hippocampal neurons [8]. It can be speculated that this early immune-mediated effect on the IgLON5 clusters induces a further intracellular pathological cascade, making later immunotherapy less effective. If true, IgG subunit analysis could reflect ongoing inflammatory activity and probably even predict the immunotherapy response. In line with this, a case with exclusively IgLON5-IgG1 inflammatory changes in the brain biopsy and MRI and a temporary response to immunotherapy has recently been reported [7].

In conclusion, early immunotherapy can be highly effective in anti-IgLON5 disease, confirming a key pathogenetic role of initial autoimmune mechanisms. Despite clinical heterogeneity, a subacute onset of characteristic symptoms, including sleep attacks and bulbar signs, should increase clinical suspicion. Being safe and non-immunosuppressive, IVIg should be tried in suspicious cases without delay.

## Data Availability

The datasets used and/or analyzed during the current study are available from the corresponding author on reasonable request.
